# Dark metabolism: a molecular insight into how the Antarctic sea‐ice diatom *Fragilariopsis cylindrus* survives long‐term darkness

**DOI:** 10.1111/nph.15843

**Published:** 2019-05-07

**Authors:** Fraser Kennedy, Andrew Martin, John P. Bowman, Richard Wilson, Andrew McMinn

**Affiliations:** ^1^ Institute for Marine and Antarctic Studies University of Tasmania Hobart 7000 Tasmania Australia; ^2^ Centre for Food Safety and Innovation Tasmanian Institute of Agriculture Hobart 7000 Tasmania Australia; ^3^ Central Science Laboratory University of Tasmania Hobart 7000 Tasmania Australia

**Keywords:** Antarctic, dark survival, *Fragilariopsis cylindrus*, metabolism, proteomics, sea‐ice algae

## Abstract

- Light underneath Antarctic sea‐ice is below detectable limits for up to 4 months of the year. The ability of Antarctic sea‐ice diatoms to survive this prolonged darkness relies on their metabolic capability. This study is the first to examine the proteome of a prominent sea‐ice diatom in response to extended darkness, focusing on the protein‐level mechanisms of dark survival.
- The Antarctic diatom *Fragilariopsis cylindrus* was grown under continuous light or darkness for 120 d. The whole cell proteome was quantitatively analysed by nano‐LC−MS/MS to investigate metabolic changes that occur during sustained darkness and during recovery under illumination.
- Enzymes of metabolic pathways, particularly those involved in respiratory processes, tricarboxylic acid cycle, glycolysis, the Entner−Doudoroff pathway, the urea cycle and the mitochondrial electron transport chain became more abundant in the dark. Within the plastid, carbon fixation halted while the upper sections of the glycolysis, gluconeogenesis and pentose phosphate pathways became less active.
- We have discovered how *F. cylindrus* utilises an ancient alternative metabolic mechanism that enables its capacity for long‐term dark survival. By sustaining essential metabolic processes in the dark, *F. cylindrus* retains the functionality of the photosynthetic apparatus, ensuring rapid recovery upon re‐illumination.

## Introduction

Primary production within Antarctic sea‐ice is spatially and temporally variable, largely due to strong physiochemical gradients within the ice and seasonal variation in solar irradiance. The light required for photosynthesis is the single most influential driver of ice‐associated microbial communities (Peters & Thomas, 1996; Arrigo, 2014), however ice typically attenuates surface irradiance from *c*. 1500 to *c*. 5 μmol photon^−1^ m^−2^ s^−1^ during the summer months (McMinn *et al*., 2010a,b). Ecological success of phototrophs relies on metabolic plasticity with respect to the pigment‐protein complexes of the photosynthetic apparatus. Ice‐associated algae must minimise damage from excess light or increase photon capture in low light by modifying the specific ratio of these complex types.

The light‐harvesting antenna in diatoms is large and, although it shares a high homology with the light‐harvesting complexes of higher plants and green algae, it differs with respect to pigment composition and excitation absorption properties (Wilhelm *et al*., 2014; Ishihara *et al*., 2015; Taddei *et al*., 2016; Giovagnetti & Ruban, 2017). It is comprised largely of the pigments chlorophyll *a* (Chl*a*) Chl*c*, and fucoxanthin, which are bound to fucoxanthin Chl*a/c*‐binding proteins (FCPs). The addition of fucoxanthin allows diatoms to absorb more of the electromagnetic spectrum, particularly the blue‐green wavelengths that dominate aquatic environments (Bertrand, 2010) Diatom genomes encode three variations of FCP, each with its own evolutionary origin and differing with respect to functionality and thylakoid interaction (Büchel, 2003; Lepetit *et al*., 2012; Nymark *et al*., 2013; Ishihara *et al*., 2015). The three clades of FCP are LHCF (those associated with all Chl*c*‐containing algae that fulfils the primary role in light harvesting), LHCR (those of distinct red‐algal origin, tightly associated with the photosystem I (PSI) antenna) and LHCx (those related to the ancient Li818 light inducible proteins of green algae that are mainly ultilised during stress (Gundermann *et al*., 2013; Wilhelm *et al*., 2014)). Variation in the type of FCP accompanying the photosystem is biologically relevant because irradiance directly influences clade expression (Lepetit *et al*., 2010; Wilhelm *et al*., 2014). Collectively, the FCPs have led to the success of diatoms in ecosystems characterised by variable irradiance, especially low‐light conditions (Nagao *et al*., 2013; Kuczynska *et al*., 2015; Morgan‐Kiss *et al*., 2016).

With the onset of the polar winter, light drops to below detectable limits for up to 4 months (Palmisano & Sullivan, 1983; Peters & Thomas, 1996; McMinn & Martin, 2013). To retain viability, polar phototrophs use strategies that modify physiology and metabolic output to maintain DNA integrity. Some taxa form cysts or resting stages, while others are facultatively heterotrophic and/or survive on stored energy reserves. Cyst and spore formation is relatively common in the Arctic, but does not appear to be a mainstream strategy among Antarctic taxa (Taylor & McMinn, 2002; McMinn & Martin, 2013). Heterotrophic uptake of organic substrates has been previously reported as a potential mechanism to supplement energy requirements (Palmisano & Sullivan, 1983; Rivkin & Putt, 1987; Laybourn‐Parry *et al*., 2005), however the energy obtained is unlikely to be sufficient to enable long‐term dark survival. Studies have also shown that the uptake and metabolism of exogenous supplied organic substrates is irregular or ineffective (Bunt & Lee, 1972; Horner & Alexander, 1972). Sea‐ice diatoms may exploit currently unknown metabolic pathways that sustain essential metabolic processes and the components required to maintain viability. The ability to rapidly regulate metabolic output in response to any given light regime would confer a competitive advantage and ensure rapid recovery of the photosynthetic machinery upon re‐illumination. Maintenance metabolism may help to explain why many sea‐ice diatoms are able to rapidly resume photosynthesis and grow rapidly after extended darkness (Peters & Thomas, 1996; Wulff *et al*., 2008; McMinn *et al*., 2010a,b; Reeves *et al*., 2011; Nymark *et al*., 2013). Despite studies indicating strong downregulation of photophysiological processes during prolonged darkness (Peters, 1996; Peters & Thomas, 1996; Popels *et al*., 2007; Wulff *et al*., 2008; Reeves *et al*., 2011; Martin *et al*., 2012), a certain level of functionality and structural arrangement must be retained in the dark. This situation infers that cells are actively maintaining some degree of photosynthetic capacity and sustaining constituents of the photosynthetic apparatus in the dark. The question remains how cells are able to sustain metabolism in the absence of a primary energy source. It is widely known that diatoms are able to use stored energy reserves in the form of lipids and carbohydrates during periods of low cellular energetics (Bunt & Lee, 1972; Smayda & Mitchell‐Innes, 1974; Antia, 1976; Peters & Thomas, 1996; Zhang *et al*. 1995; Raven and Beardall 2011; Martin *et al*., 2012; Mock *et al*., 2017) and that the rate of consumption is low. Cells might also switch to alternative unconventional metabolic pathways, or amplify the regulatory flexibility of existing pathways to enhance metabolic possibilities (Allen *et al*., 2011). For example, some marine algae use alternative electron donors and utilise fermentative metabolism under dark anoxic conditions (Fernie *et al*., 2004; Catalanotti *et al*., 2013; Kamp *et al*., 2013, 2015; Yang *et al*., 2015). Considering the complex genomic origins of sea‐ice diatoms in which alternative metabolic routes may have been acquired by the procurement of genes via horizontal gene transfer, endosymbiosis and duplication events, adjustment of metabolism via alternative routes is possible (Sims *et al*., 2006; Tirichine & Bowler, 2011; Medlin, 2016). Evidence of diatoms acquiring genes from various sources is illustrated in the functional differentiation of genes encoding isozymes of conserved metabolic functions (Falciatore & Bowler, 2002; Bowler *et al*., 2008; Smith *et al*., 2012; Mock *et al*., 2017). Smith *et al*. (2012), showed that 31% of the genes in diatoms are unique to that particular species, an observation attributed to the complex evolutionary adjustments of the genome. Some species have re‐targeted select nuclear encoded genes to discrete organelles, resulting in isozymes that target multiple subcellular locations (Kroth *et al*., 2008; Smith *et al*., 2012). For example, diatoms have approximately twice as many enzymes involved in glycolysis than green algae. A high number of isozymes can amplify regulatory flexibility and enhance the functional possibilities of metabolic processes.

The psychrophilic pennate diatom *Fragilariopsis cylindrus* is prominently ice‐associated and, as a result, has evolved specialised survival mechanisms. Therefore, it is a useful indicator species for investigations into adaptation and acclimation to the polar sea‐ice habitat (Mock *et al*., 2017). Several studies have described the effect of darkness on the survivability of Antarctic phytoplankton and sea‐ice algae (Bunt & Lee, 1972; Palmisano & Sullivan, 1983; Peters & Thomas, 1996; Baldisserotto *et al*., 2005; Reeves *et al*., 2011), but most research has focused on physiological adaptation rather than the biochemical and molecular drivers behind dark‐induced metabolism. Recent advances in functional genomics has made it possible to examine the molecular processes enabling dark survival (Nymark *et al*., 2013; Bai *et al*., 2016; Mock *et al*., 2017). In this study we aim to gain a comprehensive insight into how *F. cylindrus* can survive extended periods of darkness by examining the expression profile of the proteome. Although proteomics is still some way removed from real proof of physiological function, it does provide a unique profile of metabolic function and our results provide the first insight into the molecular basis for long‐term dark survival.

## Materials and Methods

### Algal culture

A monoculture of *F. cylindrus* was isolated from Antarctic pack ice in 2015 (Davis station, East Antarctica). This culture was taxonomically identified using the descriptions found in Scott & Marchant (2005) and confirmed by 18S rRNA sequencing (Supporting Information Notes S1). The culture was maintained in exponential growth phase in L_1_ media (Guillard & Hargraves, 1993) under cool white fluorescent light (50 μmol photon m^−2^ s^−1^, 12 h : 12 h, light : cycle) at 2°C ± 1°C in a batch system and bubbled with filtered air. Cultures were then divided into six Erlenmeyer flasks (and amended with double‐concentrated L_1_ medium; Table S1). Flasks were divided into two treatments: continuous light (50 μmol photon^−1^ m^−2^ s^−1^), *n* = 3 and continuous dark, *n* = 3. Cells were kept in their respective treatment for 120 d, with subsampling on days 0, 0.5 (12 h), 1, 3, 7, 21, 30, 60, 120. Following 120 d, dark cells were re‐exposed to 50 μmol photon^−1^ m^−2^ s^−1^ of white light for 24 h to examine cell recovery.

### DNA extraction

DNA was extracted using the boiling method developed by Liu *et al*., 2014. Firstly, the sample was heated to 95°C for 10 min in sterile 10 mM Tris pH 8.1 using 0.5 cm^2^ of *F. cylindrus* embedded on filter. Filter material was vortexed 1 min to free the maximum number of cells from the filter. After heating, the extract was cooled on ice and spun for 1 min in a microfuge. The polymerase chain reaction (PCR) was set up using MyTaq enzyme (Bioline Australia), 1 μl of template DNA, 1 μM of each primer (18S rRNA) Euk 4F 5′‐CTGGTTGATCCTGCCAG‐3′ and Euk 516R 5′‐ACCAGACTTGCCCTCC‐3′.

The thermocycling conditions used were initial denaturation at 98°C for 30 s and 32 cycles of: 95°C for 30 s, 59°C for 30 s, and 72°C for 45 s, followed by a final extension of 10 min at 72°C. The thermocycler model used was a Peltier PTC200.

Sequencing used the ABI BigDye cycle terminator kit 3.1 and an ABI 3730 xl automated sequencer (carried out at Macrogen, Seoul, South Korea). Sequences were manually checked, ambiguous nucleotide positions near the primer regions were removed, and sequence was compared with the National Center for Biotechnology Information (NCBI) database with closest sequences downloaded (SI 1).

### Proteomic sample preparation, lysis and digestion

Subsamples (100 ml) were withdrawn from each replicate and centrifuged at 10 000 ***g*** for 2 min at 2°C. The supernatant was then removed, and the resulting pellets were placed in 1 ml centrifuge tubes and spun again at 2°C for 1 min at 10 000 ***g***. The supernatant was discarded, and the resulting pellets were washed with 1 ml of ice‐cold phosphate‐buffered saline (pH 7.3) and spun at 2°C for 10 min. This step was repeated twice. The pellets were finally plunged into liquid nitrogen and stored at −80°C for *c*. 1 month before analysis.

The algal cell extracts were thawed on ice followed by the addition of 500 μl of ice‐cold high‐pressure liquid chromatography (HPLC) grade water. Extracts were then sonicated on ice for 2 min in a 100 W, 20 kHz sonicator with a titanium microtip, then plunged into liquid nitrogen. The extracts were then thawed on ice before 0.1 g of combusted quartz glass beads were added. Sample were crushed for 2 min at 2°C, and then plunged into liquid nitrogen. This step was repeated twice. Nonlysed cells and debris were subsequently collected by centrifugation at 15 000 ***g*** for 30 min at 2°C. The resulting whole cell lysates were removed and spun again at 15 000 ***g*** for 15 min to isolate any insoluble components. The supernatant was aspirated, and protein concentrations were calculated using a Pierce Coomassie Protein Assay Kit (ThermoFisher Scientific, Melbourne, Australia). Samples were normalised by taking approximately equal amounts of protein (100 μg) for each trypsin digest and analysing the same amount of each digest by LC/MS. Any remaining variation in overall signal was compensated for by the normalisation of peptide intensities across samples.

Protein solutions were added to −20°C absolute ethanol at a ratio of 1 : 9, mixed and kept at −20°C for 60 min. Samples were then centrifuged at 18 000 ***g*** for 10 min to concentrate precipitated protein. The supernatant was removed, and the resulting pellets washed with −20°C absolute ethanol. This step was then repeated. Samples were spun again at 18 000 ***g*** for 10 min; the supernatant was aspirated and the remaining pellet was left to air dry to remove any ethanol residue.

Protein samples were digested with trypsin using standard procedures (Wilson *et al*., 2010) and analysed by nanoLC‐MS/MS using an LTQ‐Orbitrap XL and Ultimate 3000 nanoHPLC system (ThermoFisher Scientific, Waltham, MA, USA). Tryptic peptides (*c*. 1 μg) were loaded onto a 20 mm × 75 mm PepMap 100 trapping column (3 mm C_18_) at 5 ml min^−1^, using 98% water, 2% acetonitrile and 0.05% TFA. Peptides were separated at 0.3 ml min^−1^ on a 250 mm × 75 mm PepMap 100 RSLC column (2 mm C_18_) at 40°C from 97% mobile phase A (0.1% formic acid in water) to 50% mobile phase B (0.08% formic acid in 80% acetonitrile and 20% water) by elution with 3–10% B over 10 min, 10–40% B over 120 min, 40–50% B over 10 min, holding at 95% B for 10 min then re‐equilibration in 3% B for 15 min. Solid phase peptide extracts were injected once and dissolved phase extracts were injected twice to achieve equivalent analysis time. The LTQ‐Orbitrap was controlled using xcalibur 2.1 software in data‐dependent mode as described in Wilson *et al*. (2016).

MS/MS spectra were searched against the *F. cylindrus* database (Joint Genome Institute, *F. cylindrus* CCMP 1102; https://genome.jgi.doe.gov/Fracy1/Fracy1.home.html) using the Andromeda search engine in maxquant, v.1.5.1.2 (http://maxquant.org/). Default settings for protein identification by LTQ‐Orbitrap MS/MS and label‐free quantitation (LFQ) included a maximum of two missed cleavages, mass error tolerances of 20 ppm then 4.5 ppm for initial and main peptide searches, respectively; 0.5 Da tolerance for fragment ions, variable methionine oxidation and fixed cysteine carbamidomethylation. The false discovery rate (FDR) for both peptide‐spectrum matching and protein identification was set to 0.01. MaxQuant peptides.txt and proteinGroups.txt output files are provided (Tables S2, S3, respectively). Proteins only identified by site, reverse sequences, contaminants and proteins identified on the basis of a single matching peptide were excluded. Peptide LFQ values, normalised according to the MaxLFQ algorithm using a minimum peptide ratio count of two (Cox *et al*., 2014), were first log_2_‐transformed and proteins identified in fewer than two biological replicates and five time points were excluded. After imputation of remaining missing values with random intensity values for low‐abundance proteins, mean LFQ values (dark vs light) were compared using a moderated *t*‐test. Protein abundance changes are expressed throughout on a log_2_ scale, in which positive values represent increased abundance in dark‐induced cells, and negative values indicate reduced abundance. Proteins with an FDR of < 2% and mean log_2_ LFQ differences >/< 0.5 (*c*. 1.5‐fold on the linear scale) were considered to be significant.

The mass spectrometry proteomics data were deposited to the ProteomeXchange Consortium via the PRIDE (Vizcaíno *et al*., 2016) partner repository with the dataset identifier PXD007098.

### Pulse‐amplitude‐modulated fluorometry (PAM)

Chl*a* fluorescence was measured using a pulse‐amplitude‐modulated fluorometer (Water‐PAM, Walz, Effeltrich, Germany) and the method described in Kennedy *et al*. (2012). Measurements were taken on days 0, 0.5, 1, 3, 7, 14, 21, 30, 60, 90 and 120 and recovery was assessed at 1, 2, 4, 8 and 24 h.

Nonphotochemical fluorescence quenching (NPQ) was calculated using the Stern–Volmer quenching parameter described in Kramer *et al*. (2004).

### Adenosine 5′‐triphosphate (ATP)

Determination of ATP concentration in cell extracts was performed through the bioluminescence of ATP with recombinant firefly luciferase and d‐luciferin (Molecular Probes, ThermoFisher Scientific) as per the manufacturer's instructions. Luminescence of samples and standards was read on a microplate reader (BMG FLUOstar Optima, BMG Labtech, Melbourne, Australia). Standard curves were conducted with each assay and the concentration of cellular ATP was normalised to cell concentration.

### Chlorophyll *a* analysis

Chl*a* concentration was determined in triplicate using the acidification protocol as described by Evans *et al*. (1987). Briefly, 20 ml subsamples were filtered onto 25 mm GF/F filters and extracted in 10 ml of methanol in the dark at 4°C for 12 h. Chl*a* extracts were subsequently measured on a Turner fluorometer (10AU Turner Designs, San Jose, CA, USA).

## Results

One of two treatments were assigned to cultures of *Fragilariopsis cylindrus*, either continuous light or continuous dark. Cells were kept in these respective treatments for 120 d, with subsampling on days 0, 0.5 (12 h), 1, 3, 7, 21, 30, 60 and 120. After 120 d, dark cells were re‐exposed to white light for 24 h to examine cell recovery. The proteomic profiles of dark cells were compared with those in constant light, this comparison revealed two key functional differences: first, widespread downregulation of proteins associated with photosynthesis and carbon fixation; and second, upregulation of respiratory processes of glycolysis, the Entner–Doudoroff pathway and the tricarboxylic acid cycle.

### Cell numbers and chlorophyll concentration

**Figure 1:**
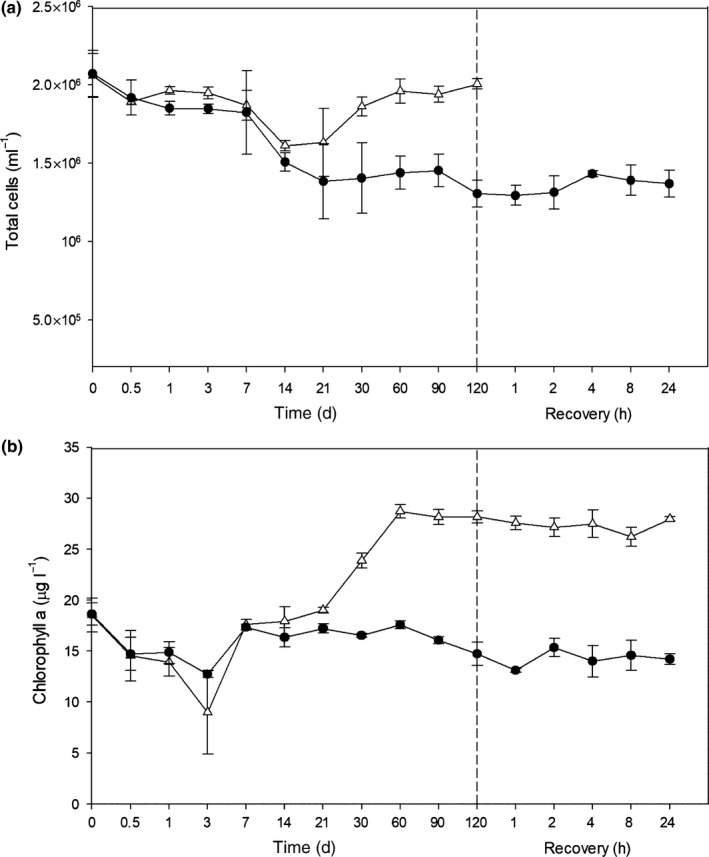
(a) Growth curves of *Fragilariopsis cylindrus* (cells ml^−1^) and (b) chlorophyll *a* concentration (μg l^−1^) of dark‐treated (closed circles) and light‐treated (open triangles) cells. Points after the dashed line represent the re‐illumination of dark‐treated cells for 24 h at 50 μmol photon^−1^ m^−2^ s^−1^. Error bars represents the standard deviation of the mean.

Cell concentration decreased in both treatments up to day 21, when stabilisation in cell numbers was observed (Fig. 1a). Numbers of cells in the constant light increased from day 30, which may indicate a degree of active growth. While proteomic samples were normalised for any changes in biomass, some minor variations in protein expressions may be present.

Following 3 d of darkness, Chl*a* concentration declined from a mean of 18.63 μg l^−1^ down to 12.74 μg l^−1^ (Fig. 1b). This concentration increased to 17.34 μg l^−1^ on day 7 and remained relatively unchanged until day 60. After which, Chl*a* decreased from 17.58 μg l^−1^ to 14.72 μg l^−1^ by day 120 of darkness. Chl*a* concentration did not differ significantly between light and dark for the first 21 d, after which Chl*a* concentration in the light increased from 19.03 μg l^−1^ on day 21 to 28.71 μg l^−1^ on day 60. Re‐exposure of dark‐treated cells to light failed to alter the Chl*a* concentration.

### Light harvesting and the photosynthetic pathway

**Figure 2:**
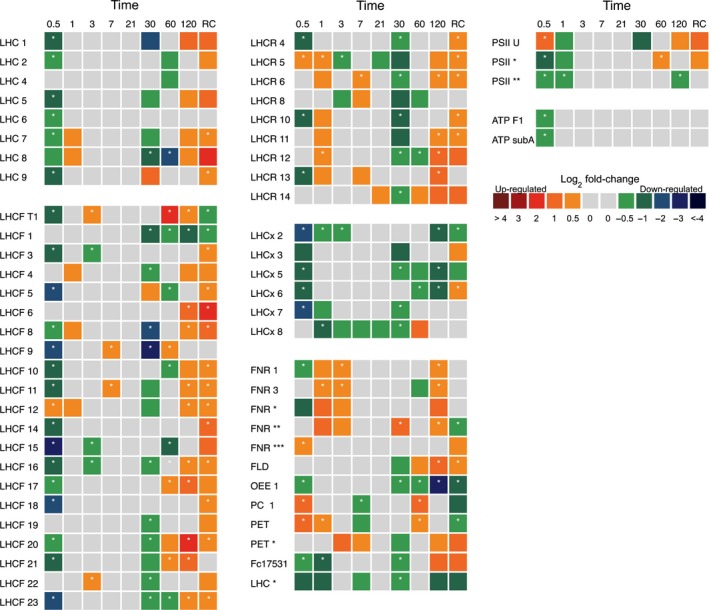
Matrix of photosynthesis‐associated *Fragilariopsis cylindrus* proteins in dark‐treated cells when compared with continuous light. Proteins are grouped by relative function and to the corresponding clade; antenna proteins (LHCF, major light harvesting; LHCR, red‐algal linage; LHCx, photoprotective; or LHC, clade unknown) or those involved in oxygenic photosynthesis. Each box represents the relative expression of the proteins indicated, after the duration of dark exposure in days, with the exception of RC, which denotes the dark recovery phase in which dark‐treated cells were re‐exposed to white light (50 μmol photon^−1^ m^−2^ s^−1^) for 24 h. The colours represent the protein abundance change (difference in mean log_2_ LFQ values, dark vs light) the scale shown. White stars (*) inside boxes represent a significant expression (FDR < 2% and log_2_ LFQ difference >/< 0.5). All expressed proteins have been normalised to relative cell abundance. Abbreviations correspond to the proteins described in Supporting Information Table S4.

Proteins from the three‐major light‐harvesting complex (LHC) clades (LHCF, LHCR and LHCx) were mostly downregulated within the initial 12 h of darkness (Fig. 2). Following this, there was limited change in the expression of these proteins in the dark when compared with the light during the first 21 d. After 30 d, downregulation of most LHC proteins across all clades was observed. In addition, oxygen evolving complex 1, photosystem II 12 kDa protein and Chl*a*/*b* binding protein Fc17531 were also downregulated. There was no change in the expression of ATP synthase subunits following 12 h. Widespread upregulation of light‐harvesting proteins after 120 d of dark exposure compared with continuous light was observed except for those classified in the photoprotective clade (LHCx), which remained downregulated. This was also reflected in proteins involved in the photosynthetic chain: ferredoxin (log_2_ +1.1‐fold), flavodoxin (+1.91‐fold), plastocyanin (log_2_ +1.3‐fold) and photosystem II 12 kDa protein (log_2_ +0.5‐fold). These were all highly expressed in the dark at 120 d. Re‐illumination of dark cells for 24 h induced significant expression of proteins in the primary light‐harvesting clades LHC and LHCF, as well as the red‐algal‐like LHCR clade. By contrast, proteins of the photoprotective LHCx clade were either downregulated or remained unchanged.

**Figure 3:**
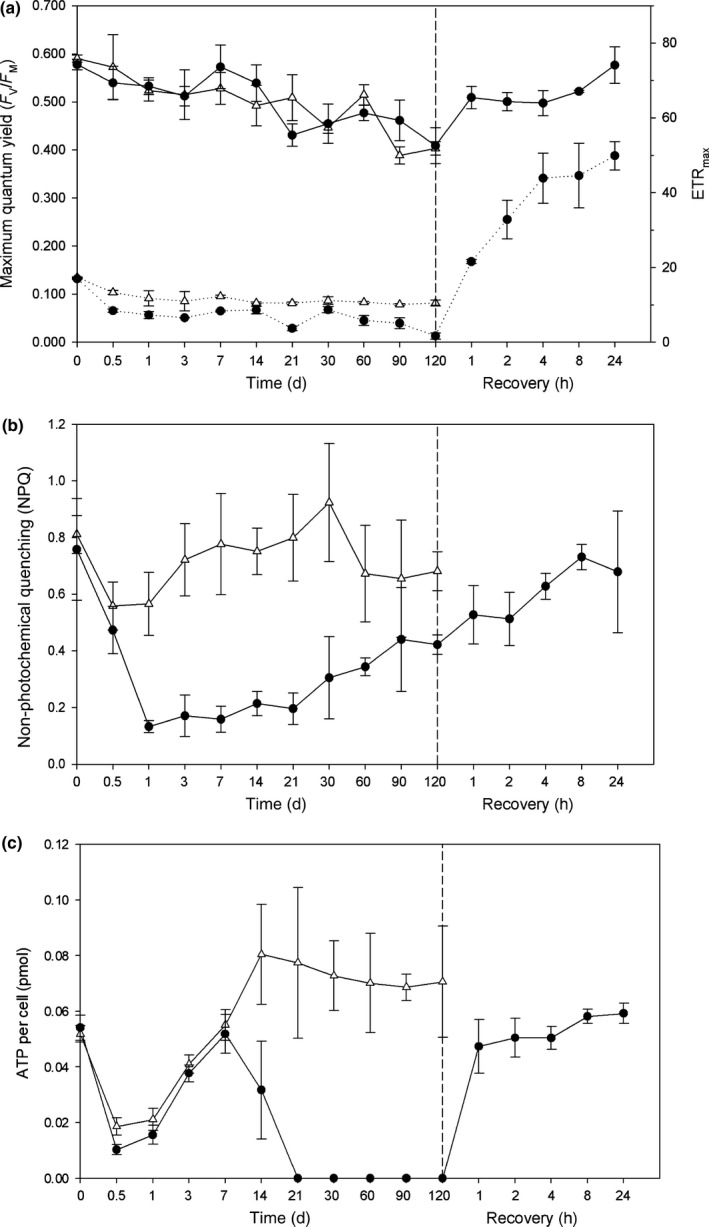
(a) Maximum quantum yield (*F*_v_/*F*_m_) and maximum electron transfer rate (ETRmax) (dotted lines). (b) Nonphotochemical quenching (NPQ). (c) ATP concentration per cell (ρmol), of dark‐treated (closed circles) and light‐treated (open triangles) *Fragilariopsis cylindrus* cells. Points after the dashed line represent the re‐illumination of dark‐treated cells for 24 h at 50 μmol photon^−1^ m^−2^ s^−1^. Error bars represent the standard deviation of the mean.

The maximum quantum yield (F_v_/F_m_) of photosystem II (PSII) was similar in both dark and light‐treated cells over 120 d (Fig. 3a). In the light, F_v_/F_m_ declined from a preincubation mean of 0.585 to a mean of 0.388 on day 120. A similar pattern was observed in the dark, F_v_/F_m_ declined from a mean of 0.577–0.461 on day 120. The maximum electron transfer rate (rETR_max_) was significantly higher (*P* < 0.05) in the light than the dark at all time points except day 14. Within 1 h of dark cells being re‐illuminated, the rETR_max_ and F_v_/F_m_ rapidly increased and continued to increase until the final assessment at 24 h, when it was well above the preincubation levels.

Non‐photochemical quenching (NPQ), which is important to dissipate adverse effects of high light intensities, was considerably lower in the dark (Fig. 3b). However, after an initial sharp decline, NPQ in the dark‐treatment group increased from 0.14 on day 1 to 0.41 on day 120. Following re‐illumination, NPQ of dark cells continued to increase with increasing light exposure. The photosynthetic parameter alpha (α) was significantly higher in the dark than the light over the course of the experiment (Fig. S1), indicating that structural breakdown between the antenna and photosystem II did not occur in the dark. Upon re‐illumination of dark‐treated cells with 50 μmol m^−2^ s^−1^ of white light, α was reduced to preincubation levels.

Cellular ATP concentration decreased to undetectable levels after 21 d of dark exposure (Fig. 2c) and remained at low levels until re‐illumination occurred. As ATP concentration is highly unlikely to be zero, the observed concentration may not be attributed to the cells current physiology but is more likely to be a limitation of the assay when ATP concentration is below the detection limit. Within 1 h of re‐illumination, cellular ATP concentrations returned to preincubation levels. ATP in light‐treated cell increased from a low of 0.018 pmol cell^−1^ after 12 h to a high of 0.080 pmol cell^−1^ after 14 d and remained at that level until day 120.

#### Pigments

While a number of proteins of the tetrapyrrole/chlorophyll biosynthetic pathway were significantly affected in dark‐treated cells, their expression changes were not co‐ordinated. The degree of expression changes and the specific roles of components in the pathway are shown in Fig. S2. Here, 3 d of darkness resulted in the sporadic upregulation of a number of proteins in the pathway, appearing to coincide with a decline in Chl*a* concentration (Fig. 1b). Limited change in expression was observed between days 7 and 21 of dark exposure. Subsequently, four out of nine enzymes in the pathway were significantly upregulated from day 30 to 120 compared with in the light. Two proteins in the pathway remained either unchanged or downregulated throughout the entire experimental period (porphobilinogen synthase (JGI|187290) and Mg‐protoporphyrin IX methyltransferase (JGI|268444)). Chl*a* increased in light‐treated cells from day 30, as a result, the protein expression in this pathway may be underestimated. The exact reason for the contrasting expression of this pathway in the dark when compared with in the light remains unclear.

Proteins of the carotenoid biosynthetic pathway were not co‐ordinately expressed in the dark when compared with in the light (Fig. S3). Phytoene desaturase (JGI|213515, JGI|270748) and ς‐carotene desaturase (JGI|229199) were predominately downregulated, whereas two violaxanthin de‐epoxidases, which are important in the generation of nonphotochemical quenching (also called diadinoxanthin de‐epoxidase) were upregulated, but at different times.

#### Calvin−Benson cycle

**Figure 4:**
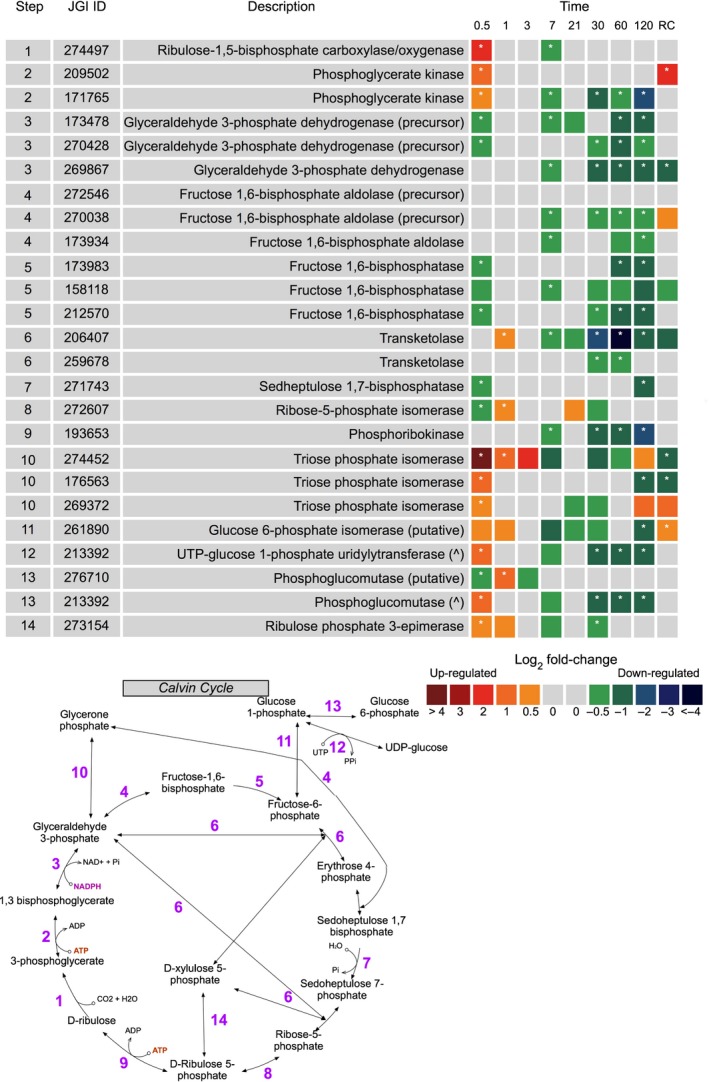
Expression of *Fragilariopsis cylindrus* proteins involved in the Calvin cycle in the dark when compared with in the light. JGI ID corresponds to the Joint Genome Institute identification number of that particular protein. Each box represents the relative expression of the proteins indicated, after the duration of dark exposure in days, with the exception of RC which denotes the dark recovery phase in which dark‐treated cells were re‐exposed to white light (50 μmol photon^−1^ m^−2^ s^−1^) for 24 h. The colours represent the protein abundance change (difference in mean log_2_ LFQ values, dark vs light) the scale shown. White stars (*) inside boxes represent a significant expression (FDR < 2% and log_2_ LFQ difference >/< 0.5). All expressed proteins have been normalised to relative cell abundance.

Most proteins involved in the Calvin cycle were downregulated in response to extended darkness when compared with in the light (Fig. 4). This was not surprising as the three main functional proteins involved: ribulose‐1,5 bisphosphate carboxylase/oxygenase (RUBISCO, JGI|274497), fructose‐1,6‐ bisphosphatase (FBPase, JGI|1739843, JGI|158118, JGI|212570) and sedoheptulose 1,7‐bisphophate (SBPase, JGI|271643) are activated by photosynthesis‐associated reactions. All proteins of the Calvin cycle were observed; widespread downregulation occurred on days 7, 30, 60 and 120. Re‐illumination for 24 h failed to alter the expression of most Calvin cycle proteins.

### Respiratory metabolism

#### Glycolysis and the Entner−Doudoroff pathway

**Figure 5:**
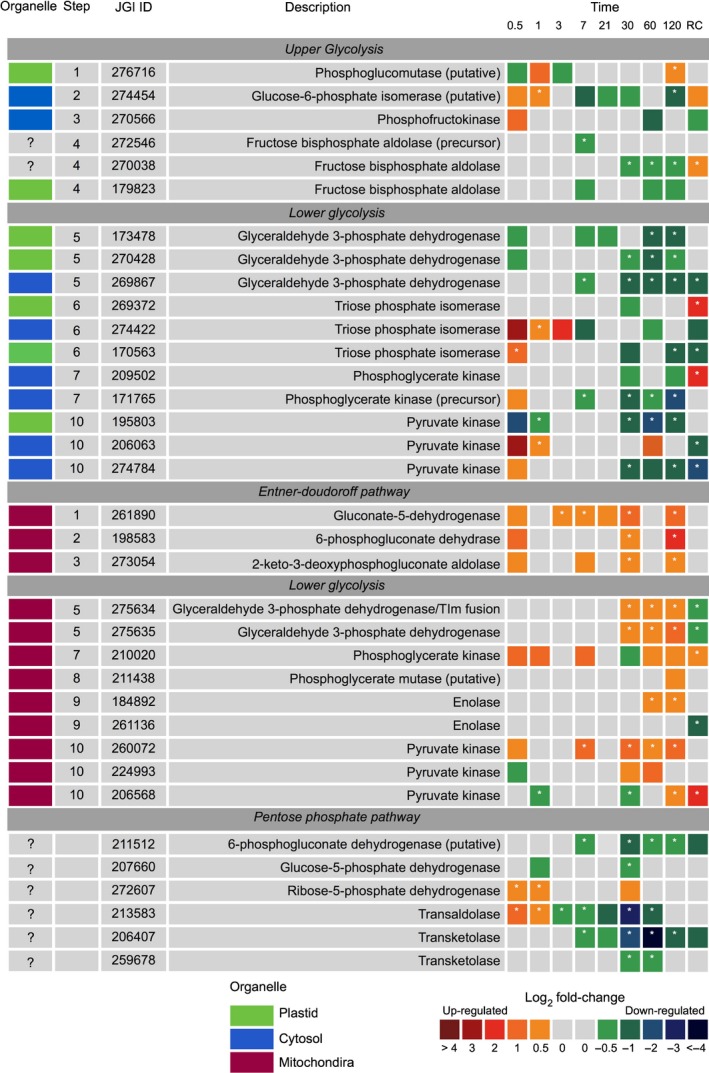
Expression of *Fragilariopsis cylindrus* proteins involved in glycolysis, Entner−Doudoroff and pentose phosphate pathways in the dark when compared with in continuous light. JGI ID corresponds to the Joint Genome Institute identification number of that particular protein. Each box represents the relative expression of the proteins indicated, after the duration of dark exposure in days, with the exception of RC that denotes the dark recovery phase in which dark‐treated cells were re‐exposed to white light (50 μmol photon^−1^ m^−2^ s^−1^) for 24 h. The colours represent the protein abundance change (difference in mean log_2_ LFQ values, dark vs light) the scale shown. White stars (*) inside boxes represent a significant expression (FDR < 2% and log_2_ LFQ difference >/< 0.5). Organelle indicates the protein suggested cellular location. All expressed proteins have been normalised to relative cell abundance.

Glycolysis occurs in two phases; the upper (preparatory) and lower (pay‐off) phases. Conventionally, the upper‐phase begins in the chloroplast and completes in the cytosol. However, all chloroplast and cytosol‐targeted glycolytic proteins were downregulated during prolonged darkness, particularly from day 30 (Fig. 5). After 30 d of darkness, some lower‐phase glycolytic proteins, thought to be mitochondrial targeted, were upregulated (Fig. 4). This change coincided with the unexpected expression of proteins involved in the mitochondrial Entner−Doudoroff pathway (EDP; Fig. 5).

Some glycolysis proteins can also function in reverse for gluconeogenesis to form glucose from pyruvate. The use of this pathway during darkness seems unlikely as the specific enzymes of the pathway were downregulated, specifically two pyruvate carboxylase (PEP) and three phosphoenolpyruvate carboxylase (PEPCK) isozymes.

The anabolic pentose phosphate pathway runs parallel to that of glycolysis. In response to dark exposure, the proteins involved in the pentose phosphate pathway were heavily downregulated compared with in the light (Fig. 5).

#### Tricarboxylic acid cycle (TCA), pyruvate and mitochondrial electron transport chain

**Figure 6:**
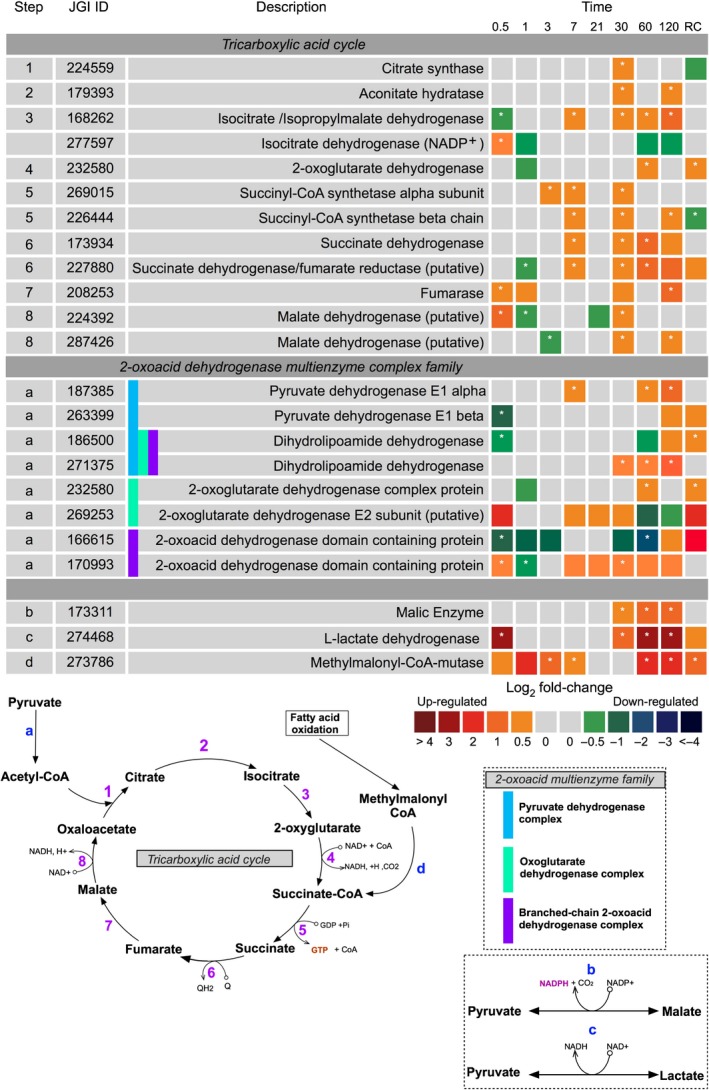
Expression of *Fragilariopsis cylindrus* proteins involved tricarboxylic acid pathway and pyruvate metabolism in the dark when compared with in the light. Steps corresponds to the stage in which a protein appears in the biosynthetic pathway. JGI ID corresponds to the Joint Genome Institute identification number of that particular protein. Each box represents the relative expression of the proteins indicated, after the duration of dark exposure in days as indicated, with the exception of RC that denotes the dark recovery phase in which dark‐treated cells were re‐exposed to white light (50 μmol photon^−1^ m^−2^ s^−1^) for 24 h. The colours represent the protein abundance change (difference in mean log_2_ LFQ values, dark vs light) the scale shown. White stars (*) inside boxes represent a significant expression (FDR < 2% and log_2_ LFQ difference >/< 0.5). All expressed proteins have been normalised to relative cell abundance.

The TCA cycle is located in the mitochondrial matrix where it catalyses the complete aerobic oxidation of pyruvate to form CO_2_, ATP, nicotinamide adenine dinucleotide (NADH), and carbon skeletons for biosynthetic processes. The TCA cycle was widely upregulated in the dark compared with the light, especially on days 30, 60 and 120. The eight main TCA cycle reactions are summarised in Fig. 6.

Imperative for TCA cycle function is a source of pyruvate. Proteins of the 2‐oxoacid dehydrogenase multienzyme family involved in the production of pyruvate were upregulated in the dark (Fig. 6). These proteins comprise the pyruvate dehydrogenase complex (PDHC), oxoglutarate dehydrogenase complex (ODHC), and the branched‐chain 2‐oxoacid dehydrogenase (BCODH).

Although specifically unrelated to the TCA cycle, l‐lactate dehydrogenase (JGI|274468) was also heavily upregulated in the dark from day 30 to day 120. This protein catalyses the reversible conversion of lactate into pyruvate. Similarly, malic enzyme (JGI|173391), which also forms pyruvate (from malate, reversible), was heavily upregulated in the dark from day 30 (Fig. 6).

Methylmalonyl‐CoA mutase (JGI|273786) was highly upregulated throughout dark exposure (Fig. 6). This mitochondrial enzyme forms succinyl‐CoA for use in the TCA cycle via the breakdown of odd‐chain fatty acids, or branched‐chain amino acids.

**Figure 7:**
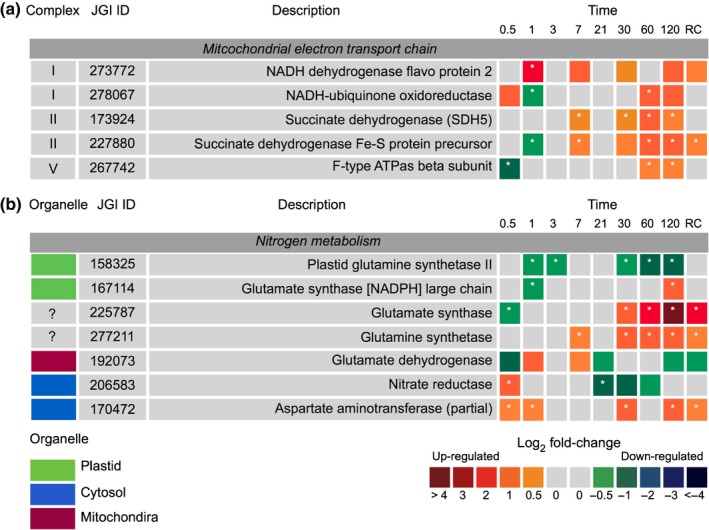
Expression of *Fragilariopsis cylindrus* proteins involved in (a) the mitochondrial electron transport chain and (b) nitrogen metabolism in the dark when compared with in the light. Complex refers to the denoted protein complex number of the transport chain, whereas organelle indicates the protein suggested cellular location. JGI ID corresponds to the Joint Genome Institute identification number of that particular protein. Each box represents the relative expression of the proteins indicated, after the duration of dark exposure in days as indicated, with the exception of RC that denotes the dark recovery phase in which dark‐treated cells were re‐exposed to white light (50 μmol photon^−1^ m^−2^ s^−1^) for 24 h. The colours represent the protein abundance change (difference in mean log_2_ LFQ values, dark vs light) the scale shown. White stars (*) inside boxes represent a significant expression (FDR < 2% and log_2_ LFQ difference >/< 0.5). All expressed proteins have been normalised to relative cell abundance.

Utilising precursors of the TCA cycle to generate energy across the mitochondrial membrane are the series of enzyme complexes of the electron transport chain (mETC). Complexes I, II and V, but not III and IV were observed. These enzyme complexes were primarily upregulated towards the latter half of dark exposure when compared with in the light (Fig. 7a).

### Urea cycle and nitrogen metabolism

**Figure 8:**
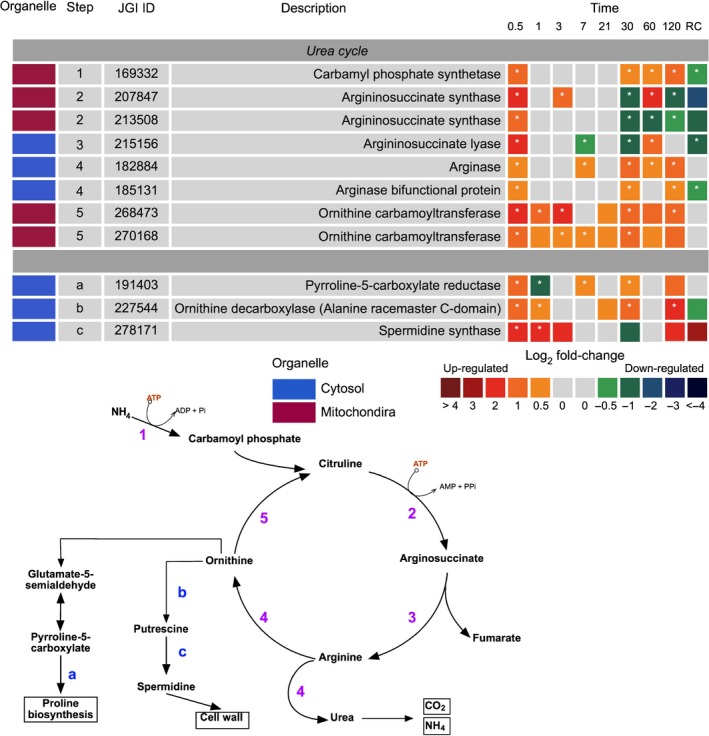
Expression of *Fragilariopsis cylindrus* proteins involved in the urea cycle and auxillaries in the dark when compared with in the light. JGI ID corresponds to the Joint Genome Institute identification number of that particular protein. Each box represents the relative expression of the proteins indicated, after the duration of dark exposure in days as indicated, with the exception of RC that denotes the dark recovery phase in which dark‐treated cells were re‐exposed to white light (50 μmol photon^−1^ m^−2^ s^−1^) for 24 h. The colours represent the protein abundance change (difference in mean log_2_ LFQ values, dark vs light) the scale shown. White stars (*) inside boxes represent a significant expression (FDR < 2% and log_2_ LFQ difference >/< 0.5). Organelle indicates the proteins suggested cellular location. All expressed proteins have been normalised to relative cell abundance.

Proteins of the urea cycle were significantly upregulated in the dark when compared with the light, but were not all co‐ordinately expressed. Argininosuccinate synthase (JGI|207847 and JGI|213508) and argininosuccinate lyase (JGI|215156) were the only proteins in the cycle to be significantly downregulated from day 30 of dark exposure (Fig. 8). All other proteins were predominately upregulated, primarily on day 0.5 and from day 30 to day 120.

The glutamate cycle (or GS/GOGAT cycle) plays a central role in the recycling and assimilation of nitrogenous compounds (Fig. 7b). Two glutamine synthase proteins were observed in the experiments; a plastid located glutamine synthase II (GSII) (JGI|158325) and glutamine synthase (GS) (JGI|277211). The former was consistently downregulated under dark conditions, while the latter was heavily upregulated from day 30. GS expression coincided with significant upregulation of a glutamate synthase (GOGAT) on day 30 (JGI|225787) and a cytoplasmic aspartate aminotransferase (AST) (JGI|170472); both are involved in the primary assimilation of ammonium. Nitrate reductase (JGI|206583), which assimilates nitrate, was downregulated from day 21 (dark).

### Fatty acid biosynthesis and β‐oxidation

Proteins involved in the synthesis of fatty acids were predominantly downregulated in the dark compared with in the light (Fig. S4). UDP‐sulfoquinovose synthase (putative: JGI|268690), which forms the important thylakoid lipid sulfoquinovosl diacylglycerol (SQDG), was upregulated in the dark from 12 h. The hydrophobic molecule carrier protein lipocalin (JGI|268796) was also upregulated in the dark from day 3. Some proteins involved in fatty acid β‐oxidation were upregulated towards the latter part of the dark incubation (Fig. S4).

### Transcription, translation and proteolysis

Ribosomal proteins were largely downregulated following 12 h of darkness (Fig. S5). After day 1, these ribosomal proteins were strongly upregulated compared with continuous light. Subsequently, there was limited change in the expression of these proteins until day 60 when a number of ribosomal 40S subunit proteins were significantly upregulated. By contrast, following 120 d of dark exposure, most ribosomal proteins were downregulated. Re‐illumination of dark cells for 24 h failed to alter the expression of many ribosomal proteins.

The downregulation of most ribosomal proteins on day 120 of dark exposure coincided with the upregulation of some serine and cysteine proteases, in addition to ubiquitin‐containing proteins and proteasome subunits (Fig. S6). Dark proteolytic subunits that are ATP dependent were predominately downregulated.

## Discussion

This study is the first to examine the proteome of a prominent sea‐ice diatom in response to extended darkness. It has been successful in illustrating the capacity of *F. cylindrus* to survive 4 months of darkness and retain photosynthetic viability. Examination of the proteome demonstrated the ability of the species to utilise cellular resources to sustain dark metabolism.

This study should be viewed with an understanding of the likely effect of constant light, which may influence protein comparisons. Cells grown in constant light are likely to have experienced oxidative stress, which can affect consumption of photosystem products via the Calvin−Benson cycle. As a result, some protein expressions may be over‐ or underestimated. Despite this caveat, this study is the first to examine the proteome of a sea‐ice diatom over an extended period of darkness and a comparison with cells in constant light was a necessary limitation. This study is intended to provide a foundation for future studies on the long‐term dark metabolism of sea‐ice diatoms.

### Light harvesting and photosynthetic pathway

Some proteins involved in light harvesting identified in this study were significantly altered in abundance during extended darkness. Within 12 h, proteins from all clades of the light‐harvesting apparatus were downregulated, with the LHCx photoprotective clade being heavily downregulated. This finding is similar to that of Mock *et al*. (2017), who also observed widespread reduction in the regulation of photosystem genes in the dark. Interestingly, these authors only observed this trend after 7 d; it remains unclear why photosynthetic proteins in this study were so rapidly downregulated. Calvin−Benson cycle proteins also exhibited a similar trend to that of photosystem proteins (dark), indicating that cells had completely abolished the photosynthetic processes in the dark. Within 1 h of light re‐exposure after 120 d of darkness, ETR_max_ exhibited higher levels following dark exposure than the pretreatment, indicating that cells regained and even improved their photosynthetic performance upon re‐illumination. A similar response in ETR_max_ following re‐illumination was observed by Nymark *et al*. (2013) and this may illustrate that the photosynthetic apparatus of *F. cylindrus* was not degraded during extended darkness. This adaptive response has been noted in previous studies with polar diatoms, in which the photosynthetic apparatus is maintained so that photosynthesis can resume rapidly upon return to favourable light conditions (Bunt & Lee, 1972; Palmisano & Sullivan, 1982; Peters & Thomas, 1996; McMinn & Martin, 2013; Schaub et al., 2017). Furthermore, if degradation of light‐harvesting antenna complexes was occurring, a gradual reduction in the photosynthetic parameter α would be apparent, as resonance energy transfer between the antenna and photosystem II would become inefficient due to physical breakdown (Falkowski & LaRoche, 1991; Vernotte *et al*., 1992; Nymark *et al*., 2013). This appears unlikely as α was maintained. Additionally, the widespread upregulation of LHCF and LHCR proteins at day 120 of dark exposure implies that cells were actively regulating and maintaining photosystem proteins.

To ensure photosystem functionality, continuous transport across multiple membranes needs to be protected and maintained. Any depletion in macromolecule transporters in thylakoid membranes would result in the degradation of the LHC. Diatom thylakoids are enriched with the anionic lipid SQDG, which accounts for over 40% of the total lipid content (Schaub et al., 2017). In this study, while speculative, the increased expression of plastid UDP‐sulfoquinovose synthase, which is essential for the production of SQDG, was observed in the dark. The potential function of SQDG in thylakoid membranes may be similar to that of higher plants and green algae in which negatively charged lipids associate and interact with plastidic ATP synthase (Pick *et al*., 1987; Joshi *et al*., 2009). In addition, the outer lamellae of low‐light‐adapted diatoms have been shown to be enriched in SQDG, ATP synthase and photosystem I (PSI) complexes (Pyszniak & Gibbs, 1992). This finding supports the concept that SQDG may act to help maintain thylakoid membrane integrity and protect the functionality of ATP synthase, although more work needs to be conducted to support this statement. Additionally, maintenance of chloroplasts in the dark may also involve the energisation of thylakoid membrane via chlororespiration. Chlororespiration has been shown to facilitate the maintenance of ATP synthase during prolonged darkness, enabling rapid ATP production upon re‐illumination (Feild *et al*., 1998; Jakob *et al*., 1999; Nixon, 2000; Lavaud et al., 2002, 2012; Wilhelm et al., 2006). The de‐epoxidation of diadinoxanthin is correlated with increased nonphotochemical quenching (NPQ) of photosystem II fluorescence. NPQ increased from day 30 (dark); this finding is consistent with the results of Jakob *et al*. (2001), who showed that de‐epoxidation of the xanthophyll cycle and subsequent increase in NPQ were highest during long‐term dark exposure in *Phaeodactylum tricornatum*. Upon re‐illumination, ATP concentration in dark exposed cells rapidly returned to pretreatment levels within 1 h and continued to increase over the next 24 h. This change not only infers active ATP synthase maintenance in the dark, but also indicates that cells may retain a fully functional LHC.

### Respiratory metabolism

Diatoms are unique in that they are the only photosynthetic organism with a mitochondrial‐targeted glycolytic pathway (Kroth *et al*., 2008; Smith *et al*., 2012). It is unknown if this is an ancient trait or one that has been recently acquired via gene transfer (Ginger *et al*., 2010). The observed downregulation of plastid/cytosolic‐targeted glycolytic proteins in the dark and the upregulation of mitochondrial lower‐phase proteins suggested significant metabolic plasticity. The physical association of glycolysis with the mitochondria negated the need to transport pyruvate and NADH into the organelle to supply the TCA cycle in the dark. Additionally, the recently discovered mitochondrial Entner−Doudoroff pathway (EDP) in diatoms can provide the necessary glyceraldehyde 3‐phosphate for the lower phase and supplement pyruvate for the TCA (Fabris *et al*., 2012). The EDP is considered to be an ancient form of glycolysis that is predominately restricted to prokaryotic lineages. EDP gene sets have are also been found in *Thalassiosira pseudonana* and *Phaeodactylum tricornutum*, seemingly obtained via horizontal gene transfer (Fabris *et al*., 2012; Chen *et al*., 2016). Regulation of the EDP has been hypothesised, as in glycolysis, to be associated with the cellular energy status with pathway flux dependent on ATP concentration (Schuetz *et al*., 2007). Cellular ATP concentration declined to undetectable levels in the dark from day 21. While speculative, this low level of ATP may have triggered the cell to initiate glycolysis and the EDP. A similar observation was noted by Fabris *et al*. (2012) in *Phaeodactylum tricornutum*, in which gene transcripts for EDP increased in the dark. Although the EDP provides less energy per molecule of glucose than the conventional glycolytic route (with net yield of 1 ATP, 1 NADH and 1 NADPH and 2 pyruvate. Glycolysis: 2 ATP, 2 NADH and 1 pyruvate), it requires less resources to synthesise the necessary enzymes (Wessely *et al*., 2011; Fabris *et al*., 2012). Coordination of several metabolic routes is derived from the necessity to conserve energy, and the move to energetic inefficiency is essentially a trade‐off between the high operational costs of alternate catabolic processes and the low investment of enzyme synthesis. Exactly why the ATP pool in the dark did not increase following activation of respiratory pathways remains unclear. This situation may imply that the rate of consumption required to maintain essential metabolic processes is equal to, or exceeds, that of production. This is highly likely if substrate level phosphorylation is the main ATP generating mechanism, as it produces less ATP than oxidative phosphorylation and is generally unable to replenish the ATP pool (Bottomley and Stewart, 1976a,b).

### The tricarboxylic acid cycle, pyruvate and the mitochondrial electron transport chain

TCA cycle proteins were co‐ordinately expressed in the dark and predominantly upregulated from day 30. This delayed upregulation appears to correspond with the expression of the EDP and lower‐phase mitochondrial glycolytic proteins. The cycle is also likely to be sustained in the dark through the action of multiple anaplerotic enzymes that act to replenish TCA cycle intermediates. These are, for example, malic enzyme to pyruvate, aspartate aminotransferase to 2‐oxoglutarate and β‐oxidation of fatty acids via succinyl‐CoA. The TCA cycle is a series of catabolic reactions that supports energy transduction, but it is also entangled in a broader network that augments multiple features of metabolism. For example, the TCA cycle has been shown to be tightly regulated with the urea cycle in diatoms (Allen *et al*., 2011). The urea cycle in this study did indeed appear to be co‐ordinated with the TCA cycle and may serve as a centre for the connection metabolism critical in balancing the demand for cellular processes when carbon fixation is abolished. A key regulatory point for TCA cycle flux, and crucial to its linkage with glycolysis, is likely to be the PDHC of the 2‐oxoacid dehydrogenase multienzyme complex family (Alejandro *et al*., 2003), but may also be regulated by thioredoxin (Daloso *et al*., 2015). While it remains unclear in this study, it is probable that thioredoxin is not a major regulator (Table S5). It is possible that the collective upregulation of the PDHC may indicate amplified acetyl‐CoA flux into the TCA cycle.

Coupled with the TCA cycle, the mitochondrial electron transport chain generates ATP in the absence of photosynthesis. The upregulation of complexes I, II and V in the dark and the corresponding expression of proteins involved in both the TCA cycle and glycolysis may indicate increased oxidative phosphorylation. This is especially apparent from day 30 of dark exposure and may highlight a specific threshold in the capacity of cells to survive in the absence of photosynthesis without mobilising reserves or inducing catabolism of nonessential metabolic components. Although when considering the observed ATP levels from day 21, any increase in abundance of oxidative phosphorylation processes must be viewed with caution, as the protein abundance of oxidative phosphorylation process in cells exposed to constant light is not regulated when photophosphorylation is the primary energy generator. This situation may result in an overestimation of the abundance of dark oxidative phosphorylation proteins, exacerbating the expression of this pathway in the dark. Metabolic flux measurements would be useful to further explore dark respiratory processes.

### Urea cycle and nitrogen metabolism

The main route of nitrogen assimilation in diatoms is via the reduction of nitrate by nitrate reductase (NR) in the cytosol. In this study, active uptake of nitrate in the dark is unlikely due to the energy requirements for assimilation and downregulation of a NR enzyme. Instead, utilisation of other nitrogenous forms may be occurring. The use of NH_3_/NH_4_^+^ formed through the catabolism of proteins and amino acids, is likely to be reassimilated through GS/GOGAT enzymes. Significant expression of enzymes involved in the catabolism of proteins and amino acids suggests heightened NH_3_/NH_4_^+^ formation. In addition, the downregulation of a plastid GSII and NADPH‐GOGAT and upregulation of GS/GOGAT with evidence for mitochondrial export, may infer heightened NH_3_/NH_4_^+^ assimilation. Tight association between the mitochondria and GS/GOGAT is essential, as it is dependent on the input of carbon skeletons in the form of 2‐oxoglutarate derived from the TCA cycle.

Similarly, associated with both pathways is the urea cycle, which in diatoms serves as a hub for recycling nitrogenous compounds (Allen *et al*., 2011; Prihoda *et al*., 2012). It is not clear why the cycle was not co‐ordinately expressed in the dark, but this finding is consistent with other studies in which alternative nitrogenous sources are available (Allen *et al*., 2011; Smith *et al*., 2016). The catabolism of proteins into their amino acid constituents provides NH_4_^+^ and monomers for TCA oxidation.

### Conclusions

The capacity for a phototroph to modify its metabolic activity to an obligatory light regime enhances its survival and success in a transient environment. Sustaining essential metabolic processes in the dark is imperative so that photosynthesis can occur rapidly upon re‐illumination. Maintenance metabolism (or the ability to live in the dark), may be one of the fundamental reasons why diatoms dominate Antarctic sea‐ice ecosystems. Our proteomic analysis of *F. cylindrus* during extended darkness has indicated that cells rapidly decrease their LHC and photosystems, while maintaining their photosynthetic capacity. This appears to be mediated through mitochondrial respiratory processes of the Entner−Doudoroff pathway, glycolysis and TCA cycle, ensuring rapid recovery upon illumination. These results align with field studies in which the ability sea‐ice diatoms to rapidly recover upon the return of light is well known. While analysis of the proteome alone is still some way removed from real proof of physiological function, it nonetheless provides a metabolic blueprint that establishes a valuable scaffold for future work to expand upon. Caveats of this study are acknowledged and, in considering these, future work requires a mechanistic understanding of the processes that are primarily responsible for dark ATP generation and ATP synthase maintenance. This would benefit from a quantitative assessment of respiration and metabolic flux, along with the molecular and environmental drivers that enable activation of the Entner−Doudoroff and mitochondrial glycolysis pathways.

## Author contributions

FK designed and performed the experiments, analysed the data, wrote the manuscript. FK, AM, AMcM and JPB planned and designed the research. RW analysed the samples.

## Supporting information

Please note: Wiley Blackwell are not responsible for the content or functionality of any Supporting Information supplied by the authors. Any queries (other than missing material) should be directed to the *New Phytologist* Central Office.

**Fig. S1** Photosynthetic parameter alpha.
**Fig. S2** Proteins involved in the tetrapyrrole/chlorophyll biosynthetic pathway.
**Fig. S3** Proteins involved in carotenoid biosynthesis.
**Fig. S4** Proteins involved in fatty acid biosynthesis and β‐oxidation.
**Fig. S5** Proteins involved in transcription and translation.
**Fig. S6** Proteins involved in proteolysis.

**Notes S1** 18s rRNA – *Fragilariopsis*_ID.

**Table S1** Medium nutrient concentrations.
**Table S2** Peptide‐spectrum matches.
**Table S3** Raw protein identifications.
**Table S4** Photosynthetic protein abbreviations.
**Table S5** Thioredoxin protein expression.
