# Supplementary material for: Dark metabolism: a molecular insight into how the Antarctic sea‐ice diatom Fragilariopsis cylindrus survives long‐term darkness
Source: New Phytol. 2019 May 7;223(2):675–91. doi: 10.1111/nph.15843 (PMC6617727; doi:10.1111/nph.15843)
Supplement: Supplementary file 1 — Fig. S1 Photosynthetic parameter alpha. Fig. S2 Proteins involved in the tetrapyrrole/chlorophyll biosynthetic pathway. Fig. S3 Proteins involved in carotenoid biosynthesis. Fig. S4 Proteins involved in fatty acid biosynthesis and β‐oxidation. Fig. S5 Proteins involved in transcription and translation. Fig. S6 Proteins involved in proteolysis. [file NPH-223-675-s001.pdf]

New Phytologist Supporting information

Dark metabolism: a molecular insight into how the Antarctic sea-ice diatom *Fragilariopsis cylindrus* survives long-term darkness

Fraser Kennedy, Andrew Martin, John P. Bowman, Richard Wilson, Andrew McMinn

Acceptance date: 2 April 2019

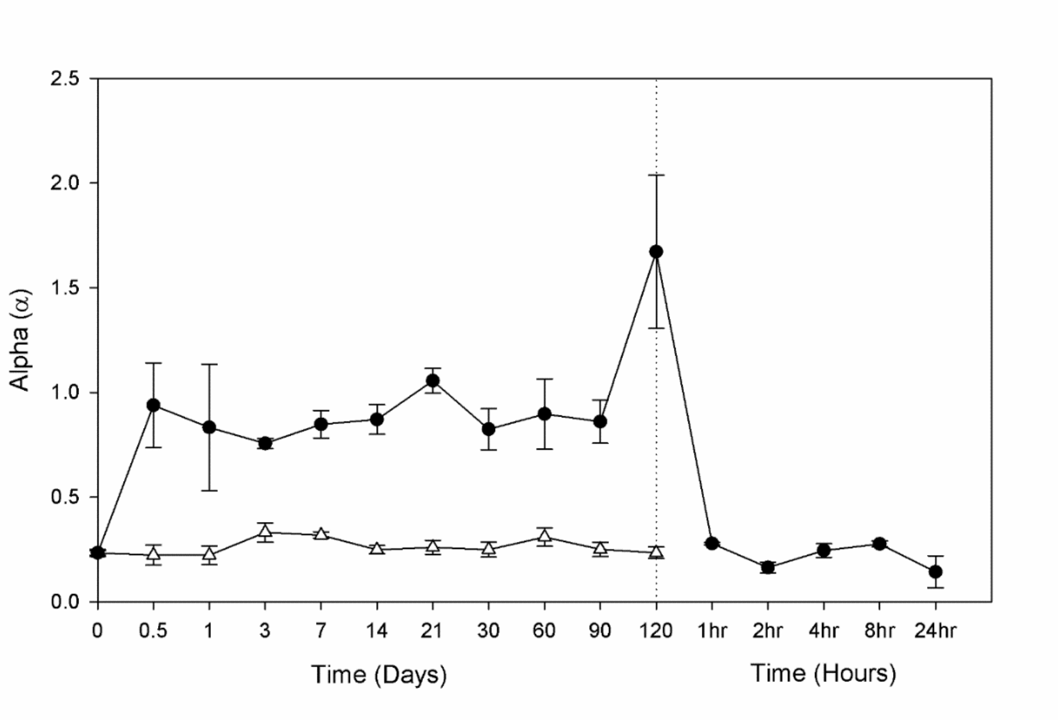

Fig. S1: Photosynthetic parameter alpha (α) of dark (closed circles) and light (open triangles) treated cells. Points after the dashed line represent the re-illumination of dark treated cells for 24 hours at 50 μmol photon^{-1} m^{-2} s^{-1}. Error bars are standard deviations of the mean**.**

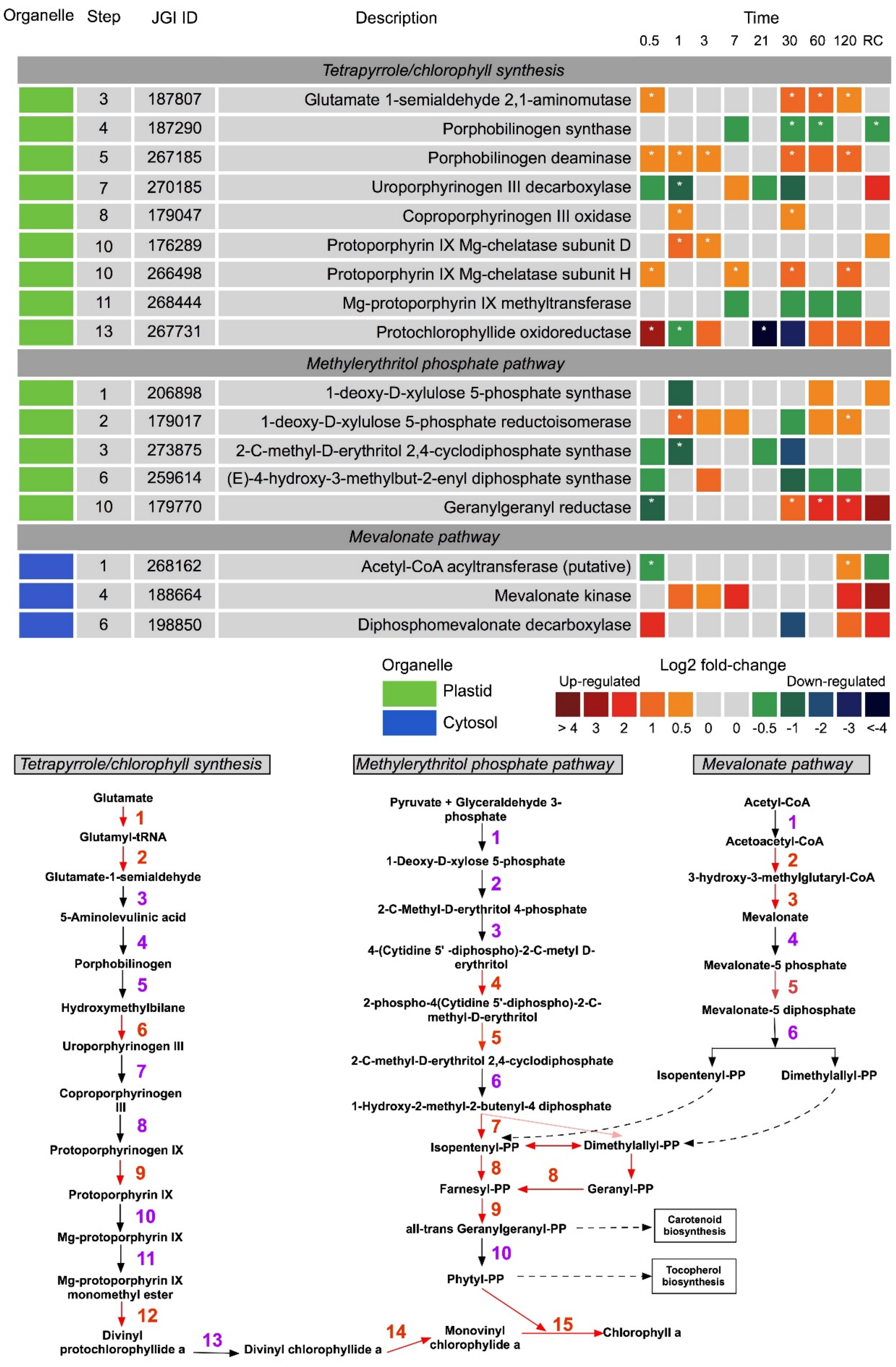

Fig.S2: Expression of proteins involved in the pathways of tetrapyyrole synthesis, methylerythritol pathway (MEP) and the mevalonate pathway (MVA) in the dark when compared to the light. JGI ID corresponds to the Joint Genome Institute identification number of that particular protein. Each box represents the relative expression of the proteins indicated, after the duration of dark

exposure in days as indicated, with the exception of RC which denotes the dark recovery phase where dark treated cells were re-exposed to white light (50 μmol photon^{-1} m^{-2} s^{-2}) for 24 hours. The colours represent the protein abundance change (difference in mean log_2 LFQ values, dark vs light) the scale shown. White stars (*) inside boxes represent a significant expression (FDR<2% and log2 LFQ difference >/<0.5).

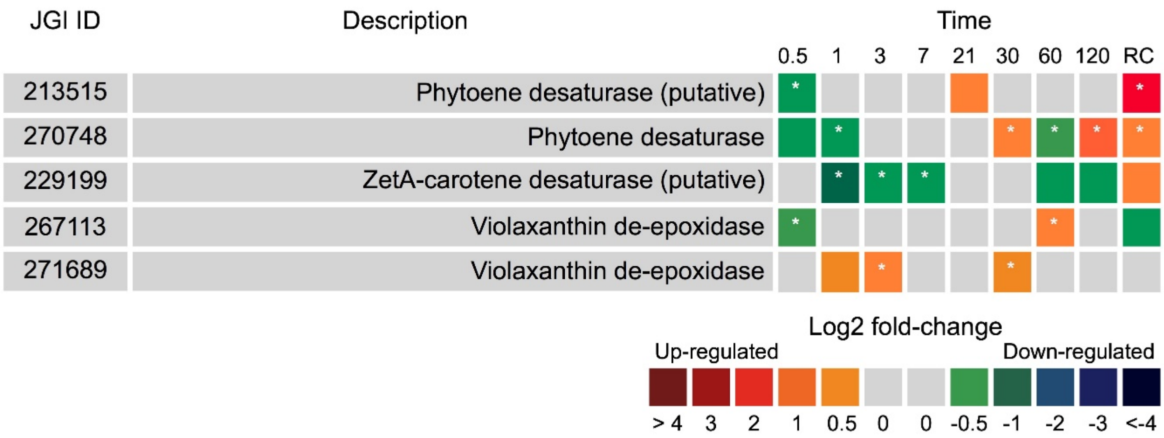

Fig. S3: Expression of proteins involved in the biosynthesis of carotenoids the dark when compared to the light. JGI ID corresponds to the Joint Genome Institute identification number of that particular protein. Coloured boxes represent the degree of expression on the log2 scale. Grey boxes indicate the protein did not display an expression above the threshold to be included. White stars (*) inside boxes represent a significant expression (<0.05 p-value). Boxes are grouped by time in days of dark exposure with the exception of RC which denotes the dark recovery phase, where dark treated cells were re-exposed to white light (50 μmol photon^{-1} m^{-2} s^{-1}) for 24 hours.

Step	JGI ID	Description	0.5	1	3	7	21	30	60	120	RC
		Fatty-acid biosynthesis									
1	274583	Acetyl-CoA carboxylase									
2	206236	Malonyl-CoA ACP transacylase									
3	227138	3-oxoacyl-synthase III									
3	227244	3-oxoacyl-synthase III									
4	228745	Beta-ketoacyl synthase									
5		3-oxoacyl-reductase									
7	273196	NAD(P)-binding protein									
8	169535	FA_desaturase_2-domain-containing protein									
-	268690	Plastid UDP-sulfoquinovose synthase									
-	268796	Lipocalin									
		Fatty-acid B-oxidation									
1	259065	Long-chain acyl-CoA synthetase									
2	209571	Acyl CoA dehydrogenase									
2	214967	Acyl-CoA dehydrogenase-like protein									
3	202663	Enoyl-CoA hydratase/isomerase						*			
4	271968	3-hydroxyacyl-CoA dehydrogenase domain									
5	268162	Acetyl-CoA acyltransferase (putative)									

(Time columns: 0.5, 1, 3, 7, 21, 30, 60, 120, RC)

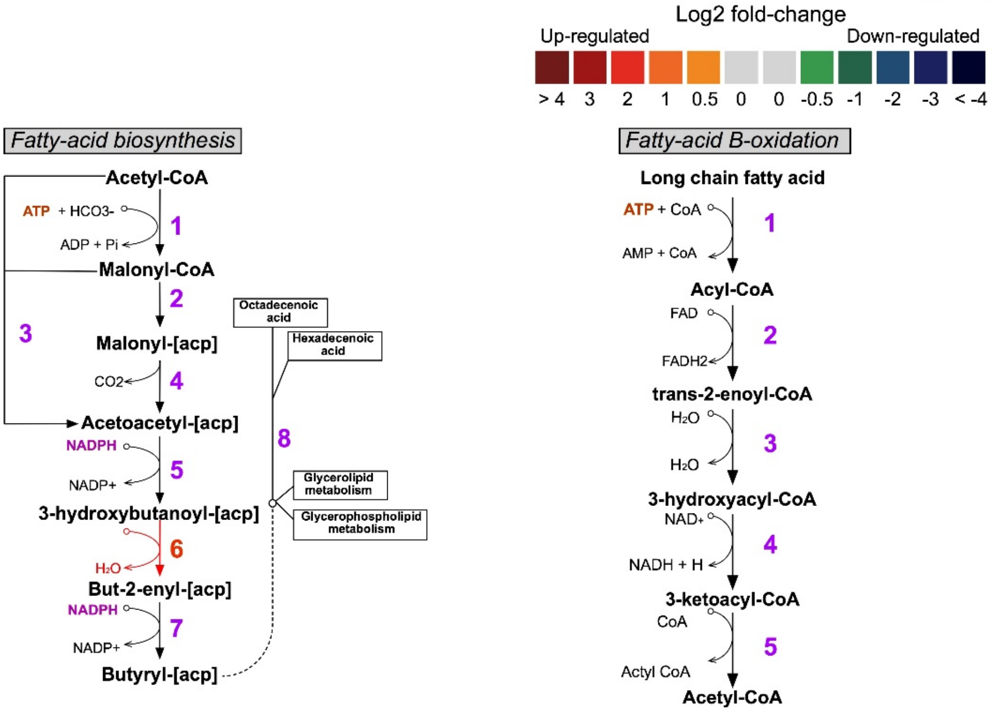

Fig.S4: Expression of proteins involved in the biosynthesis or β-oxidation of fatty-acids in the dark when compared to the light. JGI ID corresponds to the Joint Genome Institute identification number of that particular protein. Each box represents the relative expression of the proteins indicated, after the duration of dark exposure in days as indicated, with the exception of RC which denotes the dark recovery phase where dark treated cells were re-exposed to white light (50 μmol photon^{-1} m^{-2} s^{-2}) for 24 hours. The colours represent the protein abundance change (difference in mean log$_2$ LFQ values, dark vs light) the scale shown. White stars (*) inside boxes represent a significant expression (FDR<2% and log2 LFQ difference >/<0.5).

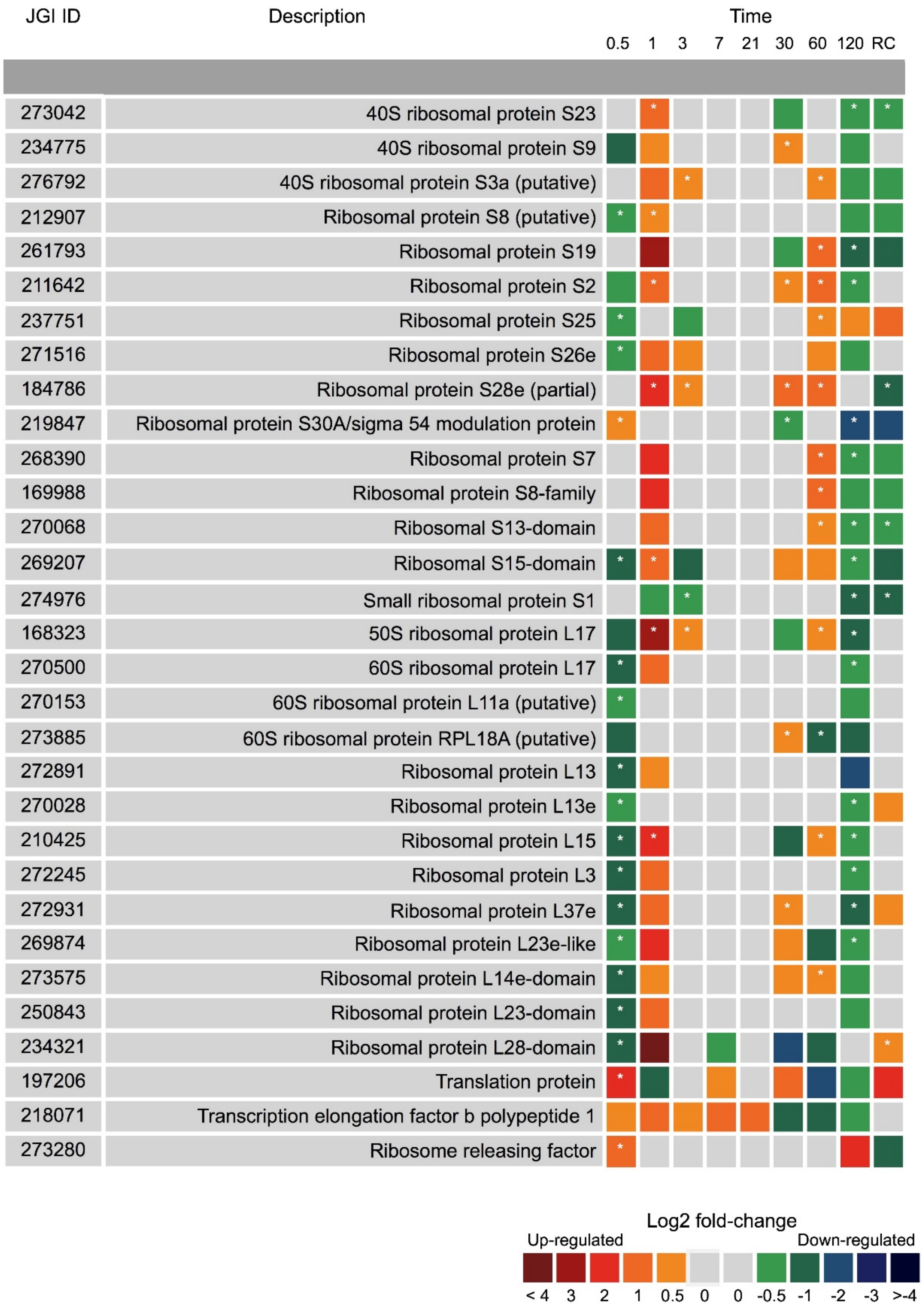

Fig.S5 Expression of proteins involved in transcription and translation the dark when compared to the light. JGI ID corresponds to the Joint Genome Institute identification number of that particular protein. Each box represents the relative expression of the proteins indicated, after the duration of dark exposure in days as indicated, with the exception of RC which denotes the dark recovery phase where dark treated cells were re-exposed to white light (50 μmol photon^{-1} m^{-2} s^{-2}) for 24 hours. The colours represent the protein abundance change (difference in mean $\log_2$ LFQ values, dark vs light) the scale shown. White stars (*) inside boxes represent a significant expression (FDR<2% and log2 LFQ difference >/<0.5).

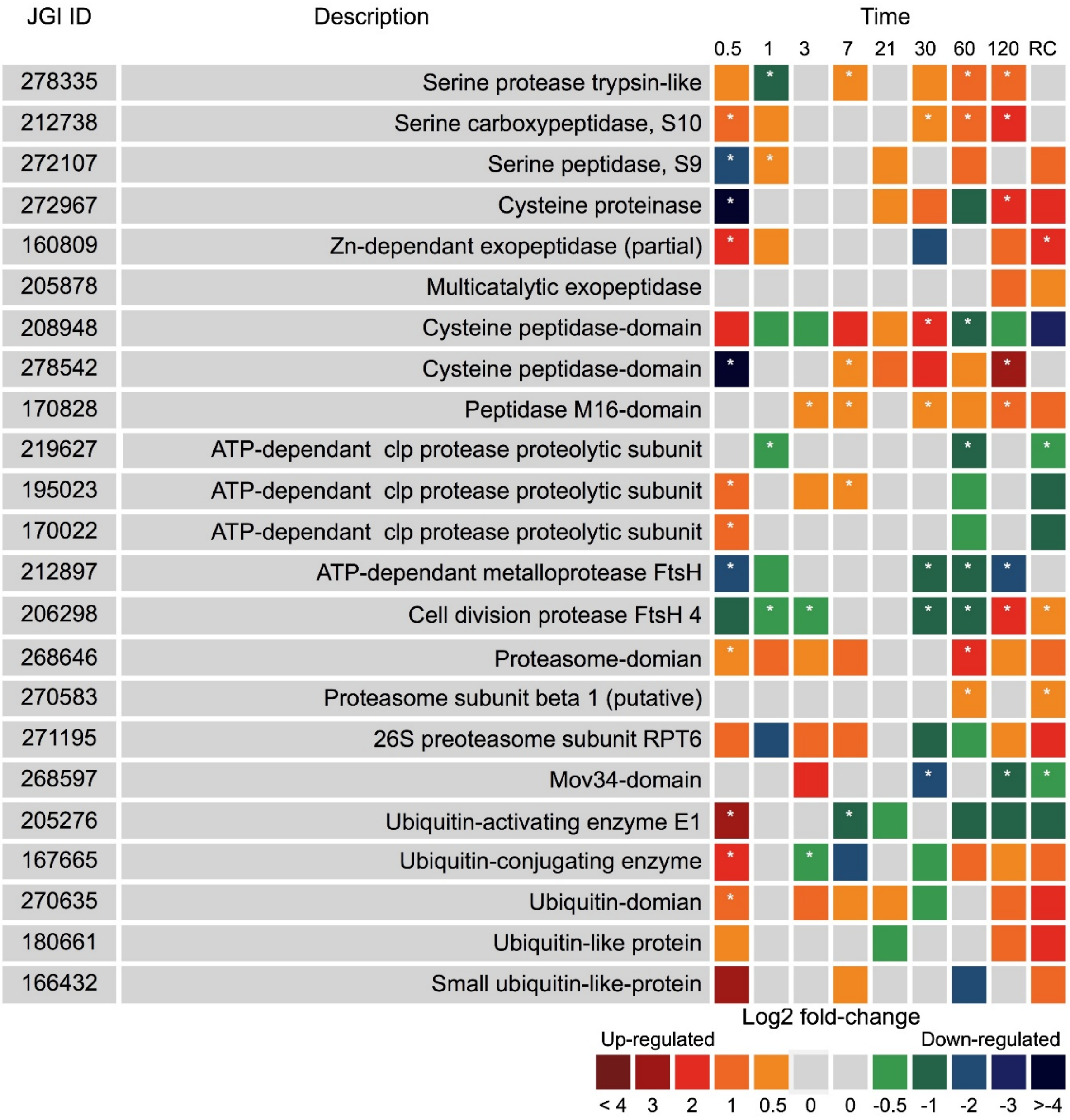

Fig.S6: Expression of proteins involved in proteolysis the dark when compared to the light. JGI ID corresponds to the Joint Genome Institute identification number of that particular protein. Each box represents the relative expression of the proteins indicated, after the duration of dark exposure in days as indicated, with the exception of RC which denotes the dark recovery phase where dark treated cells were re-exposed to white light (50 μmol photon^{-1} m^{-2} s^{-2}) for 24 hours. The colours represent the protein abundance change (difference in mean log_2 LFQ values, dark vs light) the scale shown. White stars (*) inside boxes represent a significant expression (FDR<2% and log2 LFQ difference >/<0.5).
